# A Preliminary Analysis of Sleep-Like States in the Cuttlefish *Sepia officinalis*

**DOI:** 10.1371/journal.pone.0038125

**Published:** 2012-06-06

**Authors:** Marcos G. Frank, Robert H. Waldrop, Michelle Dumoulin, Sara Aton, Jean G. Boal

**Affiliations:** 1 Department of Neuroscience, Perelman School of Medicine, University of Pennsylvania, Philadelphia, Pennsylvania, United States of America; 2 Department of Biology, Millersville University, Lancaster, Pennsylvania, United States of America; McGill University, Canada

## Abstract

Sleep has been observed in several invertebrate species, but its presence in marine invertebrates is relatively unexplored. Rapid-eye-movement (REM) sleep has only been observed in vertebrates. We investigated whether the cuttlefish *Sepia officinalis* displays sleep-like states. We find that cuttlefish exhibit frequent quiescent periods that are homeostatically regulated, satisfying two criteria for sleep. In addition, cuttlefish transiently display a quiescent state with rapid eye movements, changes in body coloration and twitching of the arms, that is possibly analogous to REM sleep. Our findings thus suggest that at least two different sleep-like states may exist in *Sepia officinalis*.

## Introduction

The Coleoid cephalopods (*e.g.* octopuses, cuttlefishes, and squids) are marine mollusks with complex nervous systems, sensory organs and sophisticated behavior [1], [2]. Cephalopod sensory systems include vertebrate-like eyes, epidermal lines analogous to fish lateral lines, vestibular systems that use analogue statocysts, and chemoreceptive organs on their lips and suckers [1], [2]. Cephalopod brains are large, multi-lobed, and complex [1], [2], and cephalopods display a wide range of learning abilities, including discrimination, spatial, and simple conceptual learning [3], [4], [5]. Cephalopods appear to use their cognitive sophistication to control their complex body patterning, used in camouflage and communication; to orient within their environments; and possibly in interactions with conspecifics [6].

Sleep has been observed in several invertebrate species [7], [8], [9], [10], [11], but only cursorily explored in cephalopods. In *Octopus vulgaris*, putative sleep states were observed that showed compensatory increases following sleep deprivation, indicative of sleep homeostasis [12]. REM sleep-like states, however, were not observed. In contrast, findings reported only in meeting abstracts indicate that cuttlefish not only sleep, but may also exhibit an analog to rapid-eye-movement (REM) sleep [13], [14], [15]. Sepia is reported to display inactive periods periodically interrupted by a state characterized by twitching of the tentacles, activation of dermal chromatophores, and possible, rapid eye movements [13], [14], [15]. The possible presence of REM sleep in Sepia is particularly interesting because REM sleep is only conclusively found in vertebrate species (birds and mammals) [16], [17], [18], [19]. Thus evidence of more than than one sleep-like state in an invertebrate may provide key insights into the evolution and possible functions of multiple sleep states.

In order to more carefully characterize sleep-like states and the possibility that cuttlefish may have more than one of them, we performed two experiments in *Sepia officinalis*. First, we determined whether or not these animals display clear rest-activity patterns and whether there appeared to be more than one sleep-like state. Second, we investigated whether rest deprivation produces compensatory increases in rest time, as is true for other animals. We find that cuttlefish display periods of inactivity similar to sleep in other animals. We also observed in a subset of adults brief periods of quiescence accompanied by phasic motor and chromatophore activity that did not appear to be wakefulness. Prolonged rest-deprivation also led to compensatory increases in quiescence, suggesting that sleep in *Sepia officinalis* is regulated by homeostatic mechanisms.

## Results

### Experiment 1

The subjects of this experiment were five laboratory-reared adult/senescent *Sepia officinalis* (15–19 months post-hatching), housed in a round tank (823 L) containing a variety of plastic plants. For the experiment, individual cuttlefish were transferred from group housing in a large (approximately 10–12 foot diameter pool) to a rectangular experimental tank (240 L). A sleeping site was prepared using a submerged clear plexiglass “V,” with the bottom point of the “V” running parallel with the length of the tank. Below the plexiglass “V” and within the narrow base of the “V” was a layer of crushed oyster shells. At each end of the “V,” two large stones were placed to create a single central resting site. Behind the tank, a few plastic plants and a large piece of black plastic served as a backdrop. A high-resolution, black and white camera (Sanyo VCB-3384, 30 frames/sec) was positioned directly above the sleeping site, and focused at a plane that maximally captured chromatophore activity on the dorsal surface of each animal. A second camera was placed facing the side of the tank.

At 8:30 AM on day 1 of the experiment, one cuttlefish was placed into the experimental tank for 2 days of acclimation. A short period of acclimation was used because these animals were very close to the end of their lifespan (most died within a few months after we began our experiments). It was fed 1 live fiddler crab (*Uca* spp); immediately thereafter, a black plastic tarp was wrapped around the entire experimental area to minimize human interaction. On the morning of day 3, the two cameras were turned on to run continuously for 48 hours. Frame data were captured on a PC with a GV-2004 video card and analyzed using accompanying software. During each minute of recording, the following behaviors were scored: ‘inactive’ (“0” buried in or lying motionless on the gravel bed) or active (“1” hovering or swimming in the water column); eyes: closed (0) or open (1); and fin movement: still (0) or moving (1). However, as the inactive/active scoring was as informative as any other single measure or combination of measures, it was used as our principle measure of rest and activity. When sleep-like states with chromatophore activation (CA; hereafter referred to as ‘sleep-like states+CA’) were observed, changes in individual chromatophore pattern components as defined by Hanlon et al [20] were tabulated second-by-second as either present or not present.

**Figure 1:**
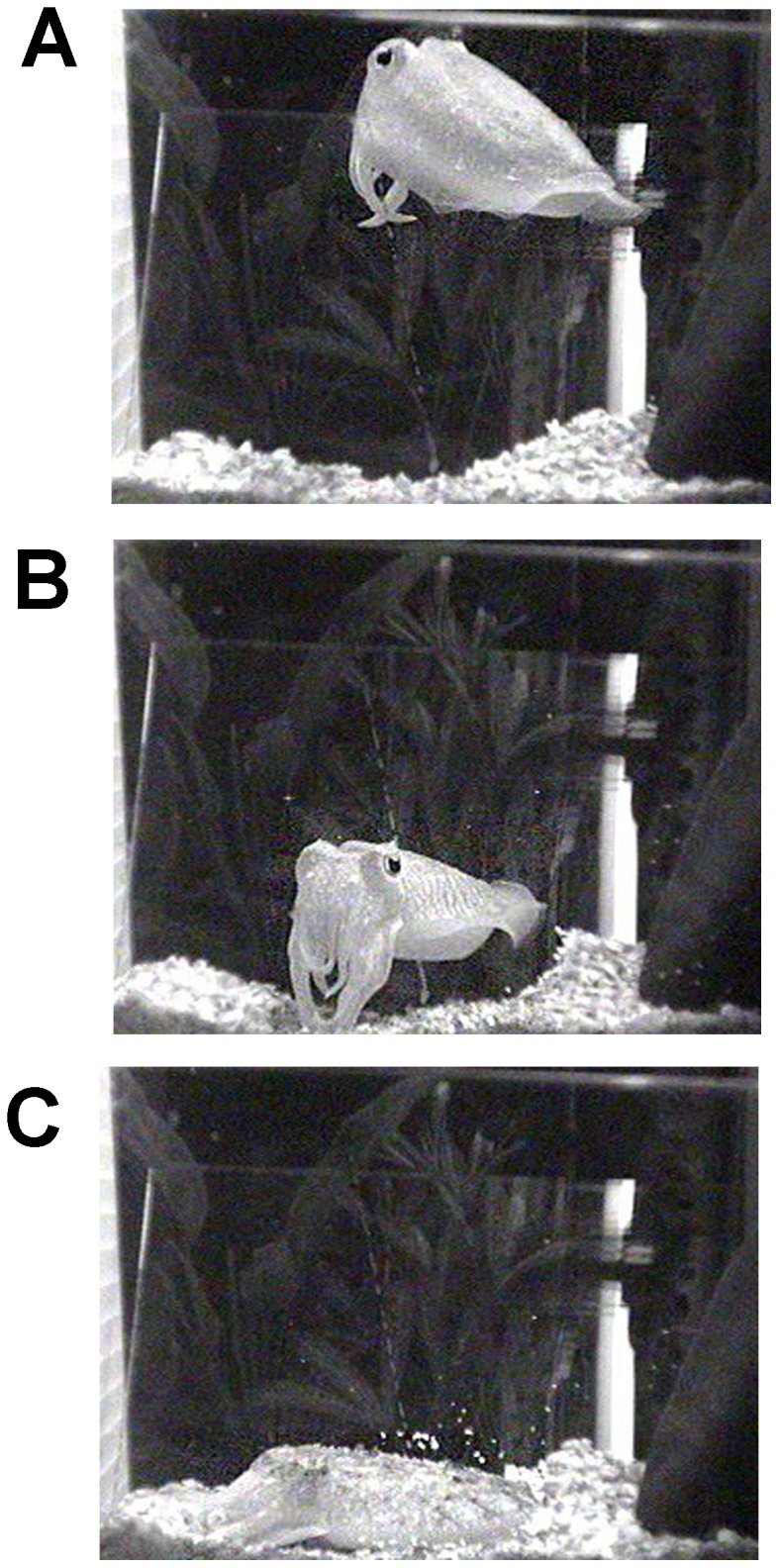
States of arousal and quiescence in the cuttlefish *Sepia officinalis*. Cuttlefish exhibit clear periods of activity where the animals actively swim (A) or hover (B) and periods of quiescence (C) where they lie on the surface or are partly buried in gravel bedding with closed eyes. An adult/senescent animal is shown.

**Figure 2:**
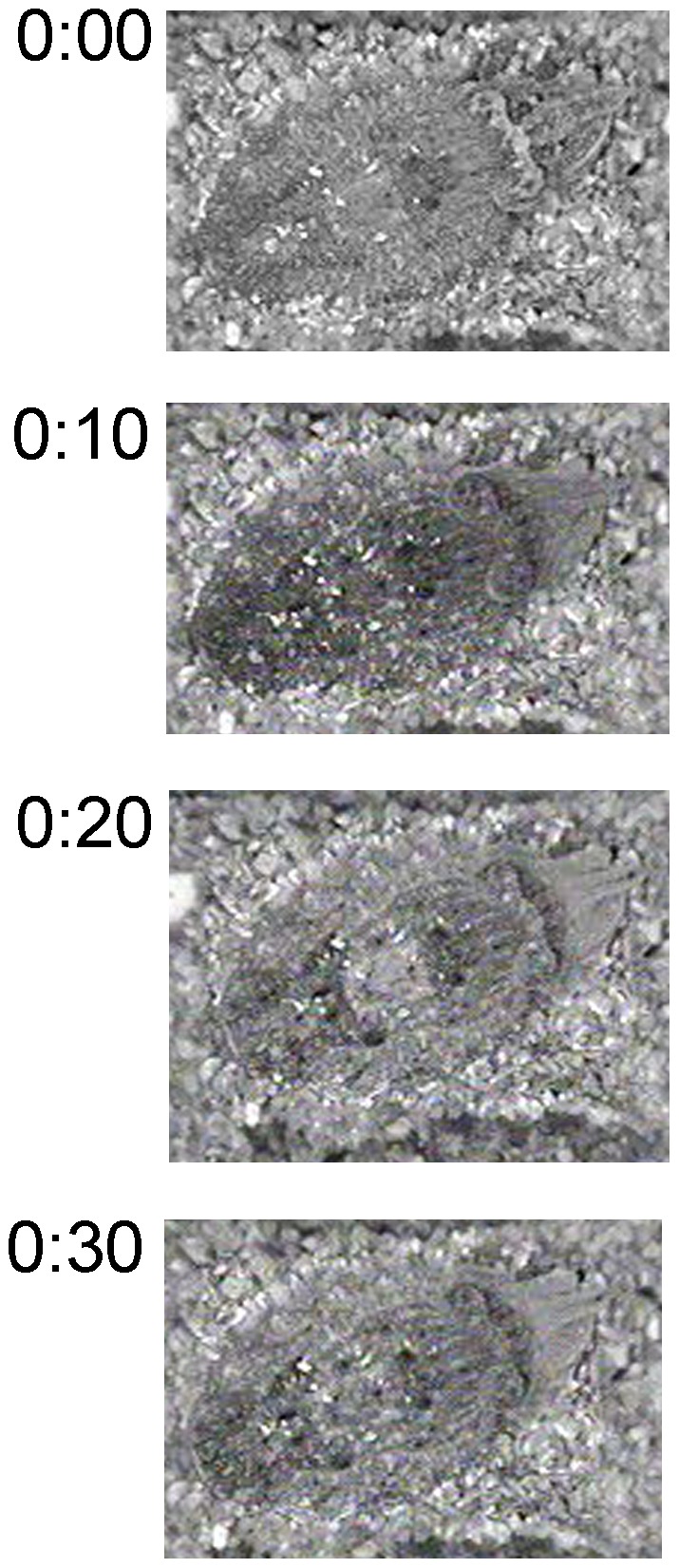
A putative sleep-like state with chromatophore activity (CA) in the cuttlefish *Sepia officinalis*. Video still frames from a representative adult/senescent cuttlefish exhibiting changing chromatophore activity during quiescence. These 4 frames show 10 second, sequential frames beginning with quiescence (0:00) and 30 seconds of the sleep-like state+CA. The camera was positioned vertically over the animal, resting on the gravel bed; the front of the animal is pointed towards the upper right corner. Changes in chromatophore components include a darkening of the body and a disappearance of a white eye-bar (0:00–0:10), a lightening of the body, the appearance of a white square (0:10–0:20) and a subsequent disappearance of the white square (0:20–0:30). Body color components as defined as described by Hanlon et al [20]. See supporting information (Videos S1,S2) for representative videos of this sleep-like state+CA.

All adult cuttlefish exhibited periods of quiescence where they rested against, or buried themselves in substrate on the bottom of the aquarium (Figure 1, Table 1). If provided enough substrate, the animals would completely bury themselves, leaving only their eyes above the gravel. This state was rapidly reversible as the animals quickly moved from the bottom and began swimming when disturbed. There was considerable variability in the amounts of rest across animals and on average there were no day-night differences in activity (Table 1). For three of the five cuttlefish, a state of complete quiescence was followed by a second, sleep-like state that contained phasic motor activity, as reported previously by Duntley et al., [13], [14], [15]. The eyes appeared to rapidly move beneath closed lids, chromatophore activity (CA) suddenly intensified, and the tips of the arms curled and twitched in a way not observed when the cuttlefish was awake (Figure 2 and Video S1,S2). This sleep-like state+CA lasted on average 135 seconds (st.dev. ±23 seconds) and only occurred once (in the night, when there was minimal illumination and disturbance) for each animal during the recording period.

**Table 1:** Quiescence in adult/senescent and juvenile *Sepia officinalis*.

	Day (% ± std.err)	Night	24-hours
Senescent (n = 5)	33±12.6	26±8	30±10.3
Juvenile (n = 4)	58.6±4.6* ‡	12.8±5.4	35.7±10

* Student's t-test, p<0.05 Day vs. night.

‡ p<0.05 senescent vs. juvenile.

The overall body patterning could best be described as mottled [20], which camouflage the animals well against mixed gravel substrates; during the sleep-like state+CA, particular components of the mottled body pattern turned on and off in an irregular sequence. These body pattern changes did not appear to be random firings of uncontrolled and uncoordinated neurons. We used two strategies to quantitatively evaluate this possibility.

**Figure 3:**
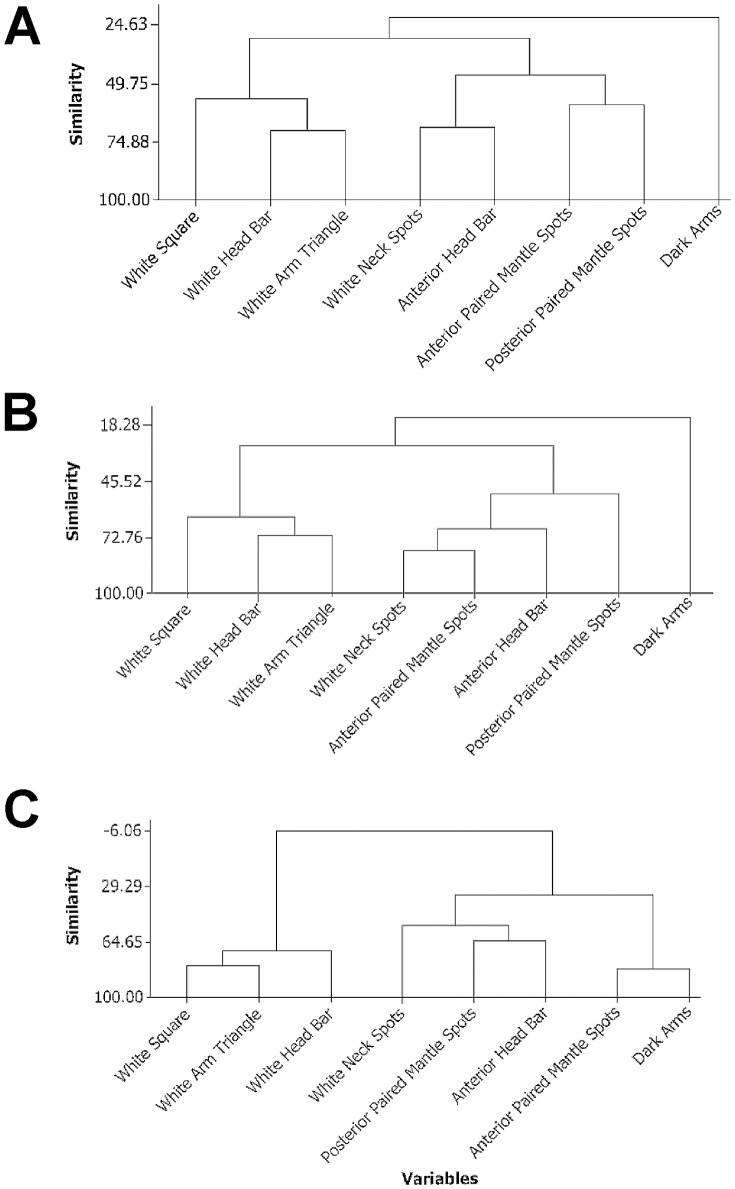
Dendrograms showing correlation coefficient distance (Ward Linkage) between body patterning components during the putative sleep-like state+CA in three adult/senescent cuttlefish (A: “C-check”; B: “Tiny”; C: “KY”). Dendrogram similarity scores range from 100% to −100% and are equivalent to Pearson or Spearman R values of “1” or “−1”.

First, we used a dendrogram analyses to determine if the chromatophore patterns observed during this state were randomly generated. Dendrograms were created using correlation coefficient distance (Ward Linkage) showing the relatedness of chromatophore pattern components (as described in [20]) during the sleep-like state+CA(Figure 3). For example, the appearances of the white square, white head bar, and white arm triangle components were positively correlated in all three cuttlefish. These components also clustered in a previous analysis of the structure of cuttlefish body patterning [21]. Similarly, white neck spots, anterior head bar, anterior paired mantle spots, and posterior paired mantle spots usually occurred concurrently with one another in all three cuttlefish. In two of the three cuttlefish (C-Check and Tiny, Fig. 3a,b), the dark arms component occurred independently and was not correlated with any other component. With the third cuttlefish (KY, Fig. 3c), the dark arms component was associated with the presence of anterior paired mantle spots.

Second, we used an autoregressive model to determine if body pattern components, taken as a whole, turned on and off at random. For each second during the sleep-like state+CA period, we determined the total number of components that were turned on as a measure of total activity. This total activity time series data was analyzed using a Box-Jenkins ARIMA model. Each of cuttlefish had a separate time series of total activity. If the body patterning components were randomly turning on and off there would be no significant autoregressive (AR) or moving average (MA) effects. Two of the three cuttlefish showed strong AR(1) time series (C-Check and KY: p<.01 for each series; p<0.10 for the third cuttlefish, Tiny) while all three showed strong MA(1) series (p<0.05 all). The moving average (MA 1) model for all three cuttlefish had a P value of less than 0.05 (C-Check: 0.00; KY: 0.001; Tiny: 0.022). Taken together, these two analyses indicate that body patterning changes were probably not manifestations of random firings of uncontrolled and uncoordinated neurons.

### Experiment 2

The subjects of this experiment were four fifth generation, laboratory-reared sub-adult cuttlefish (approximately 9 months post-hatching). An all-glass experimental tank (47 L) was divided into *ad lib* sleep and sleep-deprivation chambers. One vertical camera was positioned directly over each chamber. Both cameras collected continuous frame data throughout the experiment. A third, horizontal camera facing the sleep chamber recorded one frame every ten seconds (time-lapse). The sleep chamber consisted of a 2.5 cm layer of crushed oyster shells, one brick, and several small stones placed by the in-flow valve to prevent the cuttlefish from resting underneath the valve. A few plastic plants were placed on the outside of the tank. The sleep-deprivation chamber was free from oyster shells and small stones. A vertically-facing LCD computer monitor was positioned beneath the bottom of the tank. Hans Zimmer's “King Arthur” film score (2004) was played (Microsoft's Window Media Player) and the visualizer option was used to project random shapes on the screen. Angled mirrors placed around the sides of the tank reflected the screen image all around the cuttlefish to ensure constant, gently moving visual stimulation. Pilot studies showed that cuttlefish remained in constant motion and off the bottom of the aquarium while this stimulus was displayed.

Cuttlefish were acclimated to the sleep chamber for one week (a longer period was used as these animals were not as close to the end of their lifespan). The cameras were then turned on and baseline video data collected continuously for two days as described above. On days 3 & 4, the cuttlefish were housed in the sleep-deprivation side of the tank; during this time, the computer monitor was turned on and the CD was played. On day 5, the cuttlefish were returned to the sleep-side of the tank and video behavior was recorded for an additional 3 three days. Rest and activity were scored as in Experiment 1.

**Figure 4:**
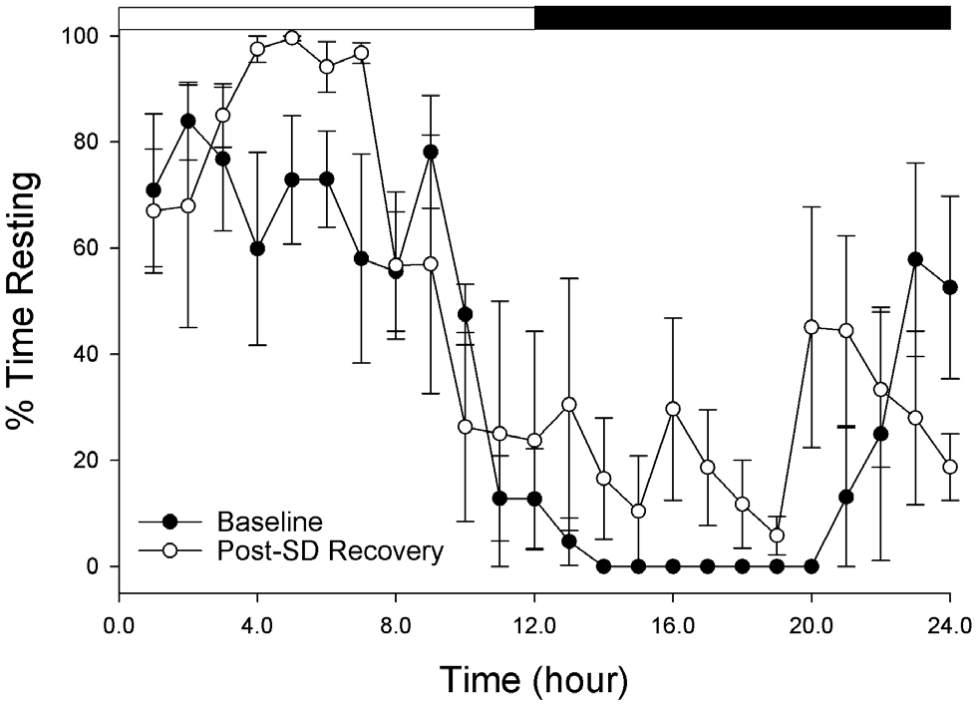
Quiescence (sleep) deprivation in the cuttlefish *Sepia officinalis*. Five juvenile cuttlefish were sleep deprived for 48 hours by continuous visual stimulation via a computer monitor positioned beneath their aquarium. During the following 24 hour recovery period, there was an overall compensatory increase in quiescence as revealed by a main effect of condition (baseline rest vs. after rest deprivation) in a 2 way ANOVA (Time and Condition as main factors): Condition: F = 6.3, p<0.013, Time: F = 9.6 p<0.001, interaction: F = 1.3, p = 0.18). Data are hourly mean (± std. deviation) behavioral scores.

In contrast to the senescent adults (Experiment 1), all juvenile cuttlefish showed clear diurnal organization in rest and activity (Table 1). Activity was highest at night, consistent with nocturnal activity patterns of wild-caught cuttlefish [22]. In addition, juvenile cuttlefish showed significantly more rest during the day than senescent cuttlefish. 48 hours of rest deprivation resulted in compensatory increases in rest time during the first 24 hours of recovery, indicative of homeostatic regulation. (ANOVA F = 6.3, p<0.013, Figure 4). None of the juvenile cuttlefish displayed a sleep-like state+CA (see Experiment 1) either before or after the 48 hours of rest deprivation.

## Methods

All cuttlefish used in the experiments were common cuttlefish, *Sepia officinalis*, hatched at the National Resource Center for Cephalopods in Galveston, Texas, from eggs obtained from the English Channel. All experiments met all standards (University, Federal, and Animal Behavior Society) for animal care and use. Water in both housing and experimental tanks was continuously recycled through a 463 liter rectangular tank that contained a particle filter, a chemical filter (activated carbon), and a biological filter (a bed of crushed oyster shells about 10.2 cm thick) that contained a large number of inoculated bacteria (thought to be *Nitrobacter* and *Nitrosomonas* spp.) to break down nitrogenous wastes. The system also included three UV-sterilizers and a chiller to maintain constant water temperature. The water was composed of Instant Ocean brand sea salts mixed into reverse-osmosis water. Water temperature was maintained at 18–20°C with salinity between 34 and 35 ppm. Room lighting was supplied by large, north-facing windows and was supplemented with three banks of manually controlled florescent lights overhead (258 LUX), that were turned on during daylight hours, approximating a 12∶12 light dark cycle. For video purposes, experimental tanks were illuminated continuously by two small sets of fluorescent lights. These lights were equipped with red filters (42 LUX) because cuttlefish cannot see red light [23]. Each cuttlefish was fed a live fiddler crab every day around 8:30 AM. Chromatophore analyses were performed using Minitab 16 Statistical Software (2010), Minitab, Inc. (www.minitab.com). The amounts of rest and activity were analyzed statistically with SigmaPlot/Stat 11.0.

## Discussion

We investigated the presence of sleep-like states in the aquatic invertebrate *Sepia officinalis*. We find that adult/senescent and juvenile *Sepia* display quiescent periods that satisfy some behavioral criteria for sleep (stereotyped posture, inactivity, rapid reversibility, diurnal rhythms [in juvenile animals]), and homeostatic regulation. A particularly interesting observation was the presence of a sleep-like state characterized by twitching of the arms, eye-movements and non-random chromatophore activity. Although sensory thresholds during quiescent states were not determined, these findings suggest that *Sepia officinalis* may exhibit two distinct sleep states. These findings are discussed in greater detail below.

REM sleep has been convincingly documented in two vertebrate orders (birds and mammals) [16], [17], [18], [19]. Consistent with unpublished results from Duntley et al., [13], we find suggestive evidence of a putative analog of avian/mammalian REM sleep in *Sepia officinalis*. Like birds and mammals, this state is characterized by phasic activation of motor circuits (against a background of quiescence) that appeared endogenous in origin, rather than exogenously driven by external stimuli. This manifests most strikingly as phasic activation of the skin chromatophores, but also includes small movements (twitches) of the arms and eyes. Intriguingly, the chromatophore activation was not random, and is thus reminiscent of the non-random nature of REM sleep motor activity. In cats, for example, brainstem lesions that remove REM sleep atonia result in stereotyped motor activity (stalking, batting at objects) during REM sleep [24]. Similar ‘oneiric behavior’ is observed in humans with REM behavioral disorder [25]. It is therefore possible that the non-random chromatophore activation observed during quiescence might be similar to patterns of activation that can occur during vertebrate REM sleep.

We also considered whether this state might instead be some type of arousal or waking-related display (i.e. defensive, mating, or hunting related). These possibilities cannot be entirely ruled out because we did not measure arousal thresholds during quiescence. However, it is unlikely that this chromatophore activation represents a waking display. In *Sepia officinalis*, defensive behavior consists first and foremost as camouflage, second as diematic body patterning (pale mantle, head, and arms, with dark false eye spots on the posterior mantle and a dark edge on the fin), and third as inking and jetting away [6], [26]. Sexual displays consist of male-male agonistic displays (zebra banding, dark eye ring, striped 4th arms, extended body posture). There are no known visual sexual displays in male-female or female-female interactions although there is some evidence that polarization of body patterning is used in recognizing conspecifics [6], [27]. With respect to hunting, cuttlefish are ‘sit-and-wait’ predators or in some cases, they may raise their first pair of arms up and their fourth pair of arms out to the side and show patterning referred to as “passing cloud” when stalking a prey item[6], [28]. None of these colorations or behaviors occurred during this sleep-like state. Nor is it likely that such displays would be made in a socially isolated animal, housed in a darkened room.

This sleep-like state with chromatophore activation and rapid eye movements was only observed in adult/senescent *Sepia*, and not in juvenile animals. This was surprising because overall amounts of quiescence were higher in juvenile animals (Table 1) and REM sleep in most mammals is more abundant during early life [29]. These findings are also inconsistent with unpublished reports from Duntley et al [13], who reported a homeostatically-regulated REM sleep-like state in *Sepia* at various ages. However, as these studies exist only in abstract form, they are difficult to evaluate and compare to our own. The significance of the late appearance of this state in *Sepia officinalis* is also unclear. It may reflect differences in acclimation between adult/senescent and juvenile animals, or differences in neural maturation. Differences in acclimation seem an unlikely explanation because the juvenile animals had much longer acclimation periods, yet they displayed no sleep-like states with CA and rapid eye movements. Differences in neural maturation seem a more plausible explanation. The central ganglia in *Sepia officinalis* necessary for many complex behaviors may not be fully mature in juveniles [30], [31]; therefore it is conceivable that the neuronal circuitry necessary for this state is only present in fully adult animals. The presence of (putative) circadian regulation could not be firmly established in both age groups. While there was clear diurnal/nocturnal organization in juvenile animals, this was not detectable in adult/senescent animals. This may reflect differences in acclimation, as pilot studies indicated that these rhythms only appeared after 1–2 weeks. However, this may also reflect an age-related attenuation of circadian regulation which is also reported in aged mammals [32].

### Concluding Remarks

An important strategy in the search for sleep function is the study of sleep in diverse animal species. Through this phylogenetic approach, scientists have discovered sleep-like states behaviorally similar to human sleep in the fruit fly [7], [33] and even nematodes [8]. These states are not only behaviorally similar to human sleep, but they may be controlled by similar molecular mechanisms [34]. Thus, the study of sleep in diverse organisms may eventually provide insights into how and why sleep evolved in the animal kingdom. This may be particularly true for REM sleep, which to date has only been consistently observed in two vertebrate orders. More studies are needed before firm conclusions can be drawn regarding the presence of sleep and REM sleep in *Sepia*. For example, we did not measure arousal thresholds in these preliminary experiments, and thus cannot confirm earlier, unpublished reports of their presence in *Sepia* [13], [14], [15]. Nevertheless, our findings provide suggestive evidence that sleep states exist in *Sepia* and that REM sleep may have evolved separately in this invertebrate animal. The ultimate cause of the evolution of such a state in *Sepia* are unknown, but it is interesting to speculate that it may be related to the relatively complex nervous system of this animal; a situation that might also explain the presence of REM sleep in birds and mammals.

## Supporting Information

Video S1 **Chronology of sleep-like behavior in representative cuttlefish ‘Tiny’.** Animal is oriented with head facing upper right corner of the frame. 0:00–1:06| Quiescence (sleep-like state), 0:07–2:09| Sleep-like state+CA (activation of chromatophores, movement of eyes beneath lids, small movements of arms, 2:10–2:45| Transitions in and out of arousal, 3:34-| Quiescence resumes.
(WMV)

Video S2 **Chronology of sleep-like behavior in representative cuttlefish ‘KY’.** 0:00–0:29| Quiescence (sleep-like state), 0:30–1:03| Sleep-like state+CA (activation of chromatophores, movement of eyes beneath lids, small movements of arms), 1:04| arousal.
(WMV)
